# The impact of different levels of oat β-glucan and water on gluten-free cake rheology and physicochemical characterisation

**DOI:** 10.1007/s13197-020-04395-5

**Published:** 2020-04-15

**Authors:** Sabina Karp, Jarosław Wyrwisz, Marcin Andrzej Kurek

**Affiliations:** Department of Technique and Food Product Development, Institute of Human Nutrition Sciences, University of Life Sciences, 159C Nowoursynowska, 02-776 Warsaw, Poland

**Keywords:** Oat β-glucan, Optimisation, RSM, Gluten-free cake, Texture

## Abstract

The demand for new gluten-free (GF) products is still very crucial issue in food industry. There is also a need for bioactive compounds and natural alternatives for food additives. For now, not only providing structure without gluten is major challenge, but also high sensory acceptance and nutritional value are on the top. This study is focused on the effect of high-purity oat β-glucan as a structure-making agent on physicochemical and sensory properties of gluten-free yeast leavened cake. The response surface methodology (RSM) was used to set the design of the experiment. Water and oat β-glucan were chosen as independent variables. Enzymatic extraction was conducted in order to obtain pure oat β-glucan (approx. 85%). Physicochemical and microstructure analyses, and a consumer hedonic test were carried out to check the quality of the final product. As a last step, verification was undertaken to compare the predicted and experimental values of the results. The results showed that the optimisation process was crucial in obtaining high-quality, gluten-free yeast leavened cake. The optimised amounts of water and oat β-glucan were 66.12% and 2.63% respectively. This proves that the application of oat β-glucan to gluten-free products is possible and gives positive results in terms of texture, volume and sensory acceptance. Due to oat β-glucan’s pro-health benefits, the final product can be seen as a functional alternative for common gluten-free products in the market.

## Introduction

Gluten-free products are currently in great demand in the food industry because of the increasing number of people with coeliac disease and other gluten-related disorders. The basic principal is to eliminate gluten from one’s diet. The sources of gluten proteins are wheat, rye and barley. Developing GF products is highly challenging because of the risk of cross-contamination with gluten cereals throughout the harvesting and production process developing GF products is highly challenging because of the risk of cross-contaminations Therefore, one of the challenges in gluten-free production is keeping it clear on every step of processing. It is problematic in already existing production lines which have to be adjusted (Naqash et al. 2017). But, the main problem that cereal technologists are working against in gluten-free bakery and pastry products is the lack of gluten, a structure making agentThe lack of gluten is the main problem cereal technologists are working against in bakery and pastry products. The main general obstacles are poor volume, texture and taste. The goal is to create structure and improve the texture of GF baked products. These products are based on rice, corn flours and different starches. Different hydrocolloids, like HPMC, guar gum and xanthan are used as gluten substitutes (Sumnu et al. 2010; Hager and Arendt 2013). Hydrocolloids can increase the viscosity of aqueous solutions via gel network formation. Some hydrocolloids are also included in dietary fibre groups. Dietary fibre is a very important ingredient that cannot be neglected in a GF diet because of its pro-health benefits. The intake of dietary fibre, however, remains insufficient. In general, a GF diet is very poor in nutrients. That is why bioactive ingredients with pro-health benefits are often added to GF formulations (Arslan et al. 2019). The main source of dietary fibre is cereals, for example oats. Oat grains are highly nutritious. Besides their high content of soluble dietary fibre, oats are a good source of antioxidants (vitamin E, phytic acid or avenanthramides). The most valuable of these, however, is β-glucan, a soluble fraction of dietary fibre that acts like a hydrocolloid. It is derived from the endosperm walls of oats or barley, and is a linear polysaccharide composed of β-(1,3) and β-(1,4) linked glucose monomers (Brennan and Cleary 2005). Cereal β-glucan can increase the viscosity of aqueous solutions via its high water binding capacity that leads to gel formation and stabilisation of the matrix. β-glucan’s technological and bioactive properties depend mainly on molecular weight (Lazaridou et al. 2004). Low and high molecular weight β-glucan has different impact on dough or batter rheology characteristics. It is connected with ability of gel formation and increasing viscosity. So, the high molecular weight β-glucan develops stronger gels and have higher water binding ability. That is why, it has larger impact on physical parameters of bakery products (rheology, firmness, specific volume). The high molecular weight β-glucan have also greater health promoting potential (Pérez-Quirce et al. 2017).These findings indicate that β-glucan could be applied in gluten-free bakery products as a structure-making component. Cereal β-glucan is considered to be a functional food ingredient in human nutrition because of its ability to reduce serum cholesterol and glycemic response (Tiwari and Cummins, 2011; Tosh 2013). Another beneficial, physiological activity of β-glucan is the ability to increase viscosity in the gut, which can modify the absorption from food bolus (Wood 2010). According to (European Commission 2009), oat is considered to be safe for most coeliacs unless it is contaminated. As for any gluten-free product, gluten content must be under 20 mg·kg^−1^. The main concern, in terms of including oats to GF diets, is proteins. Oat proteins consist of high levels of cysteine, methionine and lysine. Coeliac-safe oat proteins consist mainly of globulins, which are highly digestive with a valuable amino acid profile. Prolamines (avenins) are present in low amounts and do not contain any of the coeliac disease epitopes of other gluten-containing grains. The problem of oat contamination by other gluten-containing cereals can be solved by innovative strategies in oat harvesting and processing, as well as the selection of safe varieties based on genetic analysis (Smulders et al. 2018).

In the literature, there are few articles regarding the application of β-glucan in gluten-free baked products (Pastuszka et al. 2012; Ronda et al. 2015b; Pérez-Quirce et al. 2017). These studies were based on rice bread in which β-glucan was added as a mix of structure agents next to hydroxypropyl methylcellulose (HPMC) or a hydrocolloid blend. All of them confirmed that the addition of oat β-glucan enabled the creation of batter structure and showed the possibility of developing a sensory acceptable product that fulfils EFSA (European Food Safety Authority) (European Commission 2012) requirements regarding health claims that can be declared on the product label. Researchers, however, have also outlined the high importance of water and β-glucan content optimisation to gain a high-quality product. One of the statistical tools used to set the experimental design and perform the optimisation process is the Response Surface Methodology. As analysis of variance, RSM is also very good to observe trends in results and predict final values of parameters (Bezerra et al. 2008).

To the best of our knowledge, there is no article regarding gluten-free yeast leavened cake with oat β-glucan as the structure-making agent. In the face of consumers’ need of alternatives to gluten free bakery with long list of food additives as well as insufficient recognition of β-glucan application in gluten free batter we decided to conduct this study. Therefore, the aim of this study was to evaluate the effect of oat β-glucan on GF batter rheology and cake characteristics, and ultimately the optimisation of the addition of water and oat β-glucan in order to get the best quality of GF cake with pro-health benefits. In this study the high purity oat β-glucan, was obtained by enzymatic method that does not involve strong chemical reagents. It was prepared in powder form in order to be ready to use like other food additives. Thanks to RSM the optimized formulation of GF cake was obtained what leaded to reduce competition of water between β-glucan and starch. Those are the key points that emphasize the legitimacy and innovation of the undertaken research. From the scientific point of view, this study evaluates the possibility of replacement of protein with polysaccharide and shows what was the influence on physicochemical parameters of this replacement.

## Materials and methods

### Materials

The following raw materials were purchased in the local market—rice flour (in 100 g of flour: 6 g proteins, 78 g carbohydrates, 1 g fat, 1.4 g fibre, Melvit S.A., Warsaw, Poland), corn flour (in 100 g of flour: 9 g proteins, 70 g carbohydrates, 4 g fat, 6 g fibre, Melvit S.A., Warsaw, Poland), corn starch (Raddix, Poland), tapioca starch (Merre, Bangkok), instant-dried yeasts (Lesaffre, Marcq-en-Baroeul, France), sugar (Diamant, Poznań, Poland), salt (o’Sole, Września, Poland), rapeseed oil (Olej Kujawski, Kurszwica, Poland), hydroxypropyl methylcellulose (HPMC) (Merck KGaA, Darmstadt, Germany), eggs and water. Oat fibre powder, high in β-glucan (in 100 g: 44 g dietary fibre—23 g soluble and 21 g insoluble fraction; 16 g β-glucan; 16 g proteins, 23 g carbohydrates, 7 g fat) (Microstructure, Warsaw, Poland) was used in β-glucan extraction. Chemicals and enzymes: termamyl SC (Novozymes, Denmark), ethanol 96%, sodium phosphate monobasic dehydrate and sodium azide were obtained from Avantor Performance Materials Poland S.A. (Gliwice, Poland). The buffer solution was prepared with deionised water (Hydrolab, Poland).

### Extraction of oat β-glucan

The enzymatic extraction process was based on (Kurek et al. 2018). At first, oat fibre preparation was placed into falcon tubes and shaken for 1 h (3 g into 50 ml of water at pH 9.5) at 70 rpm in IntelliMixer RM-2 (Elmi Ltd., Latvia). Then, samples were centrifuged. The pH of the collected supernatant was adjusted to optimal value and the thermostable at 80 °C α-amylase (Termamyl SC, Novozymes, Denmark) was added. When the iodine test was negative, solutions were cooled down and 2 M acetic acid were added in order to decrease pH to 3.5. Afterwards, samples were immediately put into a hot water bath. This step enables protein denaturation and precipitation. After a second centrifugation, proteins were collected as pellets, and supernatant was poured with ethanol in a ratio of 1:1.5. Following overnight storage at 4 °C, the precipitated β-glucan was collected by centrifugation and washed on the filter with 96% ethanol. The clean gums were dried under vacuum in a vacuum oven (V500, Mammert Co., Germany) and grinded into powder. Characterisation of the obtained oat β-glucan gum was described in a previous study (Karp et al. 2019a).

### Experimental design

Two design factors—water and oat β-glucan content—were chosen as quantitative independent factors to analyse their influence on cooking yield, texture, colour, specific volume, β-glucan content and overall acceptance. The independent factors were tested at the following levels: − 1, 0, + 1, between the ranges 0.5–3% for oat β-glucan (OBG) and 30–90% for water. Those levels were established based on literature research and preliminary study where maximum and minimum doses of water and oat β-glucan were checked to see if creation of a dough structure is still possible. Using the Design Expert software version 9 (Stat-Ease, Inc., USA), 13 runs (Table 1) were calculated and the quadratic equation was chosen to describe the relations among factors and responses. The central point of the model was replicated five times. The last step was the optimisation of the formula and the experimental verification of calculated values of responses.

**Table 1:** Experimental design: amounts of oat β-glucan and water needed to be analyzed in each elaboration

Run	OBG (%)	W (%)
1	3	60
2C*	1.75	60
3C	1.75	60
4	2.63	38.79
5	1.75	90
6	1.75	30
7C	1.75	60
8	0.87	81.21
9	2.63	81.21
10	0.5	60
11C	1.75	60
12C	1.75	60
13	0.87	38.79

^*^C central points, *OBG* oat β-glucan, *W* water

### Gluten-free cake production

GF yeast leavened cakes were prepared according to the calculated combinations from the experimental design. The control sample was prepared with HPMC as a structure-making agent, while experimental samples were prepared only with oat β-glucan powder at different levels. All the basic ingredients were calculated as % of the flour-starch mix. At first, rice (41%) and corn (25%) flours, corn and tapioca starches (16.7% each), as well as HPMC (2%) or β-glucan powder, as indicated in Table 1, yeast (1.8%) and sugar (40%) were mixed with water (different levels) and eggs (25%) for 2.5 min. Then, oil (9.5%) and salt (0.3%) were added. These were mixed for another 1.5 min. Next, the batter was proofed for 10 min and divided into baking moulds. The second proofing lasted for 25 min. The conditions of proofing were 37 °C and 80% relative humidity. The samples were baked for 25 min at 160 °C. After cooling down, they were put into a plastic bag and were analysed 2 h later.

### Rheology of the batter

Rheological evaluation of the GF batter was performed on the Mars III Rheometer (Thermo Fisher Scientific Inc., USA) using plate-cone geometry. At first, the amplitude sweep test was applied to find the linear region for each sample. The range of stress was 0.1–100 Pa at 1 Hz, 10 steps were analysed at 25 °C after 5 min relaxation under 1 mm gap. The range of stress of all the samples was 0.6–10 Pa. Next, according to the method by (Ronda et al. 2015b)¸ the frequency sweep test was conducted. The applied parameters were: frequency in range 0.1–20 Hz, 1 mm gap and strain established as previously described. The test was started when 1 g of sample (without yeasts to prevent bubble formation) was put on the plate and left for 5 min. Each sample was tested in triplicate. The values of storage modulus G′ (Pa) and loss modulus G″ (Pa) registered at 1 Hz were chosen to compare the samples. Then, results were fitted to the Power Law model (1):

1 $$\tau = K\, \cdot \,\dot{\gamma }^{n}$$

where τ is shear stress (Pa), γ is shear rate (1/s), K is consistency index (Pa·s^n^) and n is flow behaviour index (–).

### Physical and technological properties of GF yeast leavened cake

#### Cooking yield and specific volume

Cooking yield was calculated using the formula below (2). Four representative cakes were weighed before and after baking and cooling.

2 $$CY = \frac{{w_{a} }}{{w_{b} }} \times 100\% ,$$

where, CY is the cooking yield (%); w_b_ is the mass of sample before baking (g); w_a_ is the mass of sample after baking and cooling (g).

The volume of the four chosen gluten-free cakes from each run was measured according to the method approved by AACCI (10–05.01) (AACCI 2000). The next step was the calculation of specific volume—the value of the volume was divided by the mass of a particular cake. Results are expressed as cm^3^/g.

#### Crumb colour

The colour of the crumbs was analysed in CIE L*a*b* system using a Minolta CR-400 colorimeter (Konica Minolta Inc., Osaka, Japan). The calibration step was done with a white reference standard (L* = 98.45, a* =  − 0.10 and b* =  − 0.13). Then, colour components were measured ten times for each run of gluten-free cakes. Lightness (L*, L = 0 black; L = 100 white), redness (− a green; + a red) and yellowness (− b blue; + b yellow) were measured with the following settings: aperture = 8 mm, standard observer 2° and illuminant D65. Additionally, the ΔE—the difference in colour—was calculated from the equation below (3):

3 $$\Delta E=\sqrt{{\left({L}_{2}^{*}-{L}_{1}^{*}\right)}^{2}+{\left({a}_{2}^{*}-{a}_{1}^{*}\right)}^{2}+{\left({b}_{2}^{*}-{b}_{1}^{*}\right)}^{2}}$$

where ∆E is total colour difference, L* is lightness, a* is redness and b* is yellowness.

#### Texture profile analysis

The texture of gluten-free yeast leavened cakes was analysed using a universal testing machine (Instron 5965; Instron, Canton, MA, USA) installed with the Bluehill 2 software. The analysis was done following Kurek et al. (2016) and Karp et al. (2019b). The double compression test was set (57 mm diameter of compression probe, speed of 120 mm/min, 50% of compression, 500 N cell capacity). Gluten-free cakes were cut into cubes (20 × 20 × 20 mm) and the test was repeated eight times for each run. Finally, only firmness (the maximum force of the first compression) was taken to the RSM analyses.

### β-glucan determination

β-glucan content was determined according to Megazyme’s (Bray, Ireland) instructions using the β-Glucan Assay Kit (Mixed Linkage). Each cake sample was dried prior to β-glucan analysis. The purity of β-glucan powder was also determined.

### Sensory acceptance

Consumer acceptance test was tested done on a previously trained group of 30 students and co-workers. They were instructed how to evaluate samples on 10-cm unstructured liking scale which started from “highly not desirable” and ended to “highly desirable”. The conditions of the sensory evaluation and all rules have already been described in Karp et al. (2016). The following crumb descriptors were rated: softness, moisture, porosity, taste, flavour and overall acceptance. The overall acceptance was chosen to RSM analysis.

### Scanning electron microscopy and computer image analysis

The structure of GF cake crumbs was observed under a scanning electron microscope (Quanta 200 scanning electron microscope, Thermo Fisher, USA). Additionally, the appearance and surface of GF cake slices was captured by the computer image analysis using Image-Pro Plus 7.0.1 software (Media Cybernetics, Rockville, MD, USA) and lamps Q IMAGING MicroPublisher 5.0 RTV (Kaiser, Surrey, BC Canada) with the colour temperature at 5400 K.

### Statistical analysis

The Design Expert software version 11 (Stat-Ease, Inc., USA) was used to set the experiment, analyse the effects of the independent variables on the chosen responses and generate the 3-D surface plots. The quadratic equation described the model. In order to check the model’s accuracy, the lack-of-fit test and the coefficients of determination (R^2^) were also applied. The differences between treatments were checked by one-way analysis of variance and Fisher’s test (Statistica version 13, StatSoft, Inc., Tulsa, OK, USA). The following parameters (responses) were chosen for the RSM analysis: cooking yield, firmness, L*, ΔE, specific volume, β-glucan content, overall acceptance. Specific volume and overall quality were specified as the maximum desirable, while firmness and ΔE as the minimum. The last step of RSM was the optimisation process and verification. The predicted values of responses were compared with those experimentally established. The control sample contained only HPMC as the structure-making component. Significant differences were analysed using ANOVA with applied Fisher’s test at p ≤ 0.05.

## Results and discussion

### Rheological characterisation of gluten-free batter

The rheological properties of gluten-free batter determine its quality after baking. The determination of the viscoelastic properties of the batter is a key factor. In this study, the oscillatory test was applied. Storage (G′) and loss (G″) modules at 1 Hz were taken into consideration during the analysis of results obtained from the frequency sweep test. Finally, those results were fitted to Power Law model (R^2^ ranged between 0.820–0.992, with one exception 0.334) to explain the viscoelastic behaviour of GF batters with different levels of OBG and water. The results are presented in Table 2. The flow behaviour index (n) can take different values—n = 1 means the Newtonian law, n < 1 means shear-thinning flow (pseudoplastic behaviour) and n > describes shear-thickening flow behaviour (Fischer et al. 2009). In this study, values of n for most of samples ranged between 0.069 and 0.505 (no significant differences) that shows pseutoplastic behaviour—viscosity decreases with increasing shear rate. This founding is in line with (Bozdogan et al. 2019) who carried out experiment about the effect of quinoa flour on GF batters. The consistency index (K) ranged between 0.007–8079.5 (Pa s^n^) (significant differences). If we look at the same water level it is clearly seen that if the content of OBG increase, the values of K also increase. It means that OBG improved viscous behaviour of batter, what proves structure-making properties of OBG. The values of both modules differed significantly and were affected by water and β-glucan content. The highest values (ranging between 6994.5–5652.1 Pa for G′ and 5426,50–3062.01 Pa for G″) were noted for samples where the content of water was very low (30% or 38.79%), while the content of β-glucan was either very low (less than 2%) or quite high—at the level of 2.63%. Those samples were more consistent than the control sample. The lowest values of G′ and G″ were observed in samples where the content of water was high (81.21–90%) and the content of β-glucan was low (0.5–1.75%). These outcomes are in line with Ronda et al. (2013), who examined the influence of dietary fibres on the rheological behaviour of gluten-free dough. Batters where the content of β-glucan was higher and the level of water was lower were more consistent. The explanation behind this is that, as a hydrocolloid, β-glucan binds water and creates a gel-like network (Brummer et al. 2014). This network retained the other ingredients of the formulation (e.g. starch) that can lead to structure formation in the cake after baking. β-glucan competes with other molecules for water; therefore, it is necessary to optimise the addition of water and β-glucan. Otherwise, β-glucan could bind to too much water, which would lead to a solid structure and very consistent batter. In our previous study (Karp et al. 2019b), we characterised correlation between rheology (modulus G′, G″) and physical properties (firmness, springiness, cohesiveness, porosity and specific volume) of gluten-free cake with high-in-oat β-glucan fibre powder. It was observed that the higher values of G′, G″ are, the better textural properties are. Those findings are in line with those above.

**Table 2:** Rheological characterization of gluten-free batters: the mean values and standard deviation of storage (G′) and loss (G″) modules at 1 Hz and results of curve fitting to power law model

OBG (%)	W (%)	G′ (Pa)	G″ (Pa)	K (Pa s^n^)	n (–)	R^2^
Control		5278.50 ± 684.50^d^	1993.01 ± 268.00^c^	4904.32 ± 331.97^d^	0.263 ± 0.01a	0.981
3	60	1690.00 ± 302.01^c^	890.35 ± 215.65^b^	1338.51 ± 29.19^bc^	0.267 ± 0.03a	0.956
1.75	60	831.45 ± 59.15^b^	463.67 ± 71.65^e^	897.45 ± 115.74^bc^	0.269 ± 0.03a	0.942
2.63	38.79	6994.50 ± 232.50^f^	5426.50 ± 1336.50^e^	8079.50 ± 265.01^f^	0.348 ± 0.04a	0.951
1.75	90	72.13 ± 11.15^a^	52.91 ± 10.48^a^	59.67 ± 9.42^ab^	0.069 ± 0.02a	0.334
1.75	30	6140.50 ± 450.51^e^	3633.50 ± 226.51^d^	6460.43 ± 179.61^d^	0.313 ± 0.05a	0.953
0.87	81.21	0.56 ± 0.36^ab^	5.37 ± 0.33^a^	0.0068 ± 0.01^a^	4.337 ± 0.81b	0.989
2.63	81.21	594.25 ± 114.15^ab^	326.65 ± 44.35^ab^	572.05 ± 63.55^b^	0.332 ± 0.06a	0.992
0.5	60	188.91 ± 50.40^ab^	125.45 ± 30.55^a^	495.45 ± 88.50^b^	0.505 ± 0.04a	0.820
0.87	38.79	5652.01 ± 878.05^de^	3062.01 ± 228.00^d^	5246.12 ± 280.42^d^	0.328 ± 0.04a	0.976

Letters (a–e) show the significant differences in a column (p ≤ 0.05)

*OBG* oat β-glucan, *W* water

### Checking the model’s adequacy

The adequacy of the model was checked and validated—there was no lack-of-fit, each R^2^ value was higher than 0.90. Two independent variables—water and β-glucan content—were taken into consideration in order to optimise their addition to GF yeast leavened cake. Table 3 shows the regression coefficients obtained after fitting the model. The interaction between variables and responses was presented in linear, intercept and quadratic terms.

**Table 3:** Regression coefficients of predicted quadratic polynomial models for physical sensory and β-glucan values in GF yeast leavened cake with oat β-glucan

Factor	CY (%)	Specific volume (cm^3^/g)	L*	ΔE	Firmness (N)	β-glucan conent (g/100 g)	Overall acceptance
constant	2.12	2.12	69.44	1.73	4.40	1.07	7.99
OBG	0.09NS	0.09*	0.59*	− 1.01**	− 0.38*	0.56**	0.57NS
W	− 0.12**	− 0.11**	− 2.57**	− 0.26NS	− 0.42NS	0.12NS	0.26NS
OBG^2^	− 0.09NS	− 0.09*	0.54NS	0.25NS	− 0.57NS	0.19NS	− 0.08NS
OBG × W	0.12NS	0.12*	1.92**	− 0.89NS	− 0.50**	0.23NS	0.95*
W^2^	− 0.34*	− 0.33**	1.31**	1.35**	0.40NS	− 0.10NS	− 1.31**
R-SQ	92.9	94.9	93.7	91.2	90.1	90.5	92.3
Lack of fit	0.985	0.987	0.981	0.991	0.983	0.991	0.978

*OBG* oat β-glucan, *W* water, *NS* no significance, *R-SQ* adjusted square coefficient of the fitting model, *CV* coefficients of variation

^*^Significant at p ≤ 0.05

^**^Significant at p ≤ 0.01

### The influence of water and oat β-glucan addition levels on chosen responses

**Fig. 1:**
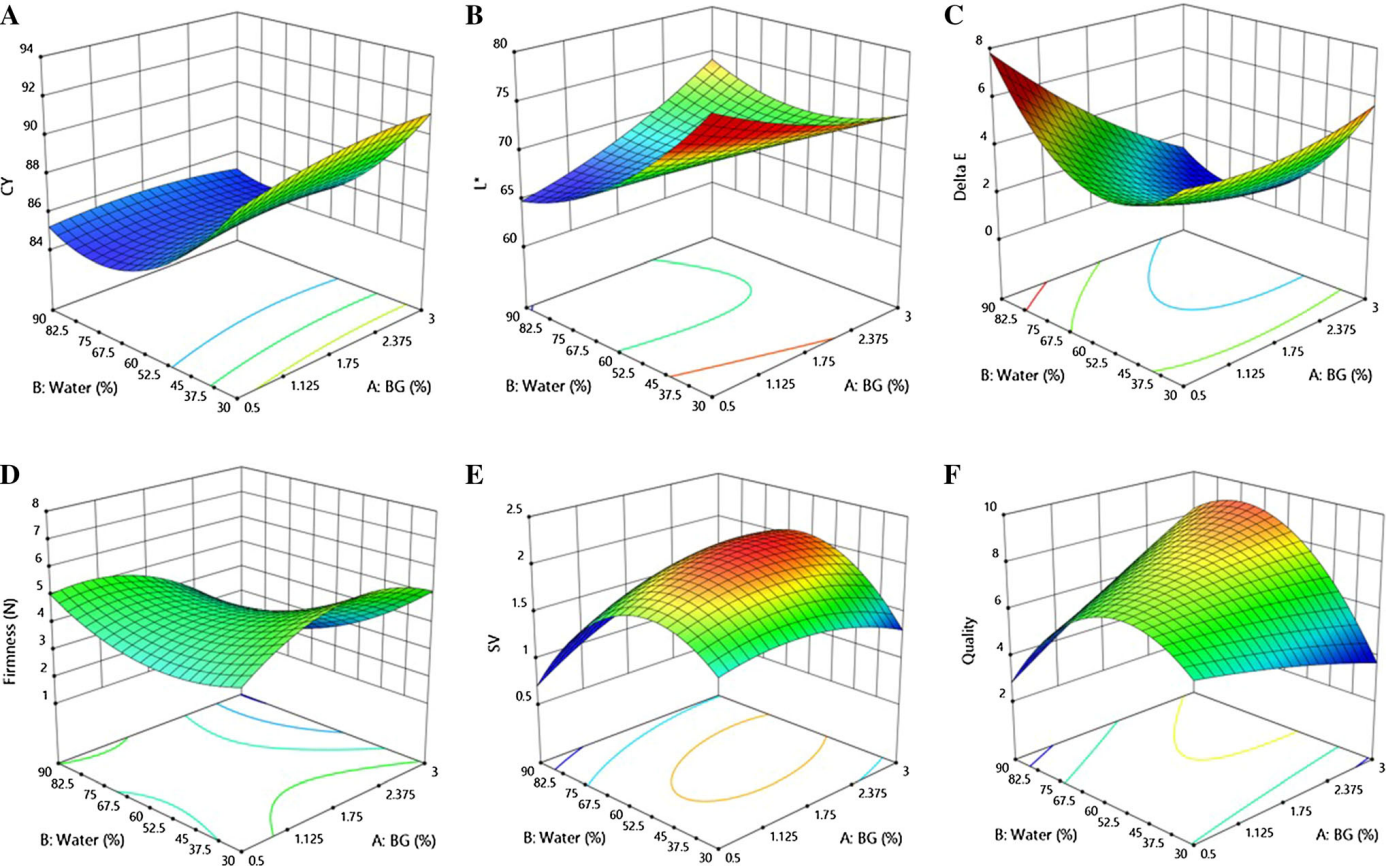
The 3-D surface plots obtained in model. Responses: **a** cooking yield (CY), **b** lightness (L*), **c** difference in color (ΔE), **d** firmness, **e** specific volume (SV), **f** overall acceptance (quality)

The RSM was used to evaluate the effect of water and oat β-glucan on GF batter and to observe trends of changes in results when independent variables had different values. Based on regression coefficients (Table 3) and the 3-D surface plots (Fig. 1), it can be said that both independent variables had significant influence on GF yeast leavened cake at different levels depending on the response. Concerning the cooking yield (CY), it was observed that only water content had significant influence on this parameter and CY decreased by adding more water in linear and quadratic terms (p ≤ 0.01 and p ≤ 0.05 respectively). The increase of β-glucan slightly increased the CY in linear terms but this effect was statistically non-significant. In comparison to other studies, there is no strong evidence as to whether cooking yield was increasing or decreasing, especially since differences were not significant (Pastuszka et al. 2012; Hager et al. 2011). Specific volume, however, was significantly affected by OBG in linear terms (p ≤ 0.05), and the linear relation of water and OBG (p ≤ 0.05). This signifies that the simultaneous impact of both variables resulted in the increase of volume, which was highly desirable. Other relations were also significant. Nevertheless, the water content, especially in quadratic terms, decreased the specific volume significantly (p ≤ 0.01 and p ≤ 0.05). A similar tendency was observed in quadratic terms with the OBG addition, where slight decrease of the specific volume was noted (p ≤ 0.05). These findings are in line with other studies, where specific volume was dependent on the concentration of OBG. Ronda et al. (2015a, b) found that the highest amount of OBG (3.9%) had the lowest specific volume, while medium (2.6%) concentration had the highest value of specific volume (2.4 and 2.56 mL/g respectively). What is more, the same tendency was observed, for linear and quadratic terms, for OBG and water. Lightness (L*) was chosen to analyse one of the colour components. This parameter was significantly dependent on both water and OBG (p ≤ 0.01 and p ≤ 0.05 respectively). While OBG tended to increase L*, water tended to decrease L*. The linear interaction of these variables, however, showed an increasing tendency (p ≤ 0.01). The significant increase of lightness was also observed in quadratic terms for water content (p ≤ 0.01). Another response connected with the crumb colour of GF cakes was the difference in colour—ΔE. This was significantly influenced by OBG addition in linear terms (p ≤ 0.01), which means that the higher the addition of OBG, the less visible the difference between the samples. The second significant impact on ΔE was water in quadratic terms (p ≤ 0.05). This indicates that when the amount of water was higher, the difference in colour was also less visible. In comparison to the experiment conducted by Duta and Culetu (2015), where they added oat brans to gluten-free cookies, lightness was decreasing with the increasing addition of oat brans. Also, ΔE was significant. It shows that the pure gum of OBG is better because it almost does not affect the colour of the product. As concerns firmness, the influence of OBG was significant (p ≤ 0.05), as well as the interaction between water and OBG (p ≤ 0.01). Those factors decreased firmness, which means that the cakes were softer—a positive result. Other interactions were not significant. Sabanis et al. (2009) compared maize, oat and barley dietary fibres. The results showed that the addition of oat fibre caused the decrease of firmness of gluten-free bread. Another study, where the optimisation process (RSM) of oat and barley β-glucan in gluten-free bread was also investigated, showed that OBG tended to increase firmness but that the linear interaction between β-glucan and water was decreasing this parameter (Ronda et al. 2015a). To sum up this section, the results of physical properties can be also compared to study of Hager et al. (2011). They investigate the influence of two soluble dietary fibres—inulin and oat β-glucan on wheat and gluten-free dough. They also analysed bake loss, texture, specific volume, colour, moisture content, microstructure, rheology and also staling rate during 5 days period. The experiment showed similar findings to those obtained in our study. The main conclusion says that OBG is more suitable for gluten-free applications that to wheat dough. Next, the adjusting of water level is key element to gain high quality product where negative effects are limited as much as it is possible. OBG decreased values of firmness, so the crumb was softer, but the specific loaf volume was also decreased. The decrease of lightness values and improvement in dough elasticity was also observed. What is more, addition of oat β-glucan reduced stale rate and decreased bake loss. Those results are connected with high water binding capacity of oat β-glucan. And because there is no gluten network, oat β-glucan do not compete with other components that much. And as a consequence, the development of crumb structure is done. There is an opposite effect in wheat dough. And finally, application of oat β-glucan definitely increased nutritional value of gluten free breads.

In addition to physicochemical analysis, a consumer acceptance test was also carried out. Overall quality was chosen as a response to RSM. The increase of this parameter was caused by the simultaneous action of OBG and water (significant at p ≤ 0.05). The quadratic terms of the effect of water, however, showed that the more water was added, the overall quality decreased. The study of Pérez‐Quirce et al. (2014) also underlined that the optimised amount of water and β-glucan is important in terms of sensory quality and acceptance. As Capriles and Arêas (2013) and Sabanis et al. (2009) revealed, the addition of dietary fibre (inulin, oat, maize, barley) improved the overall acceptance of gluten-free baked goods.

### Verification of optimisation of gluten-free batter

The last step of RSM was verification of the optimised additions of water and oat β-glucan. In order to obtain the best quality product, the responses were set as follows: firmness and ΔE as minimum, while β-glucan, specific volume and overall quality were specified as maximum. The predicted values of responses were empirically verified. The optimised dosage of water was calculated as 66.123% and OBG as 2.634%. In Table 4, the results of optimisation and verification are presented. Predicted and experimental values were comparable almost in every case. The difference was in firmness—the experimental value was higher than the one predicted. In comparison to the control sample (6.84 N), however, both were much lower (3.21 N and 4.76 N respectively). The obtained value of the specific volume for the optimised sample was almost the same as for the control sample (2.03 and 2.02 cm^3^/g respectively). This result confirms the ability of OBG to create the desirable structure of GF cake that is comparable to the structure obtained with commonly used food additives. The most positive outcome was the significant increase of β-glucan content in the optimised sample and the highest score in overall acceptance.

**Table 4:** Optimized addition of water and oat β-glucan

Design factors	Optimum amount in formulation (%)
β-glucan	2.634
Water	66.123

Responses	Predicted values	Experimental values	Control
CY (%)	85.09^a^	86.29^b^	85.88^b^
L*	70.49^a^	71.74^b^	70.68^a^
ΔE	0.76^a^	1.13^b^	1.31^b^
Firmness (N)	3.21^a^	3.56^a^	6.84^b^
Specific volume (cm^3^/g)	2.09^a^	2.03^a^	2.02^a^
β-glucan (g/100 g d m)	1.91^b^	2.01^b^	0.28^a^
Overall acceptance	7.92^a^	8.38^b^	7.64^a^

The comparison of predicted values of responses from the model and experimental values to control sample

^*^Letters (a, b) show the significant differences in a row (p ≤ 0.05)

### Scanning electron microscopy

**Fig. 2:**
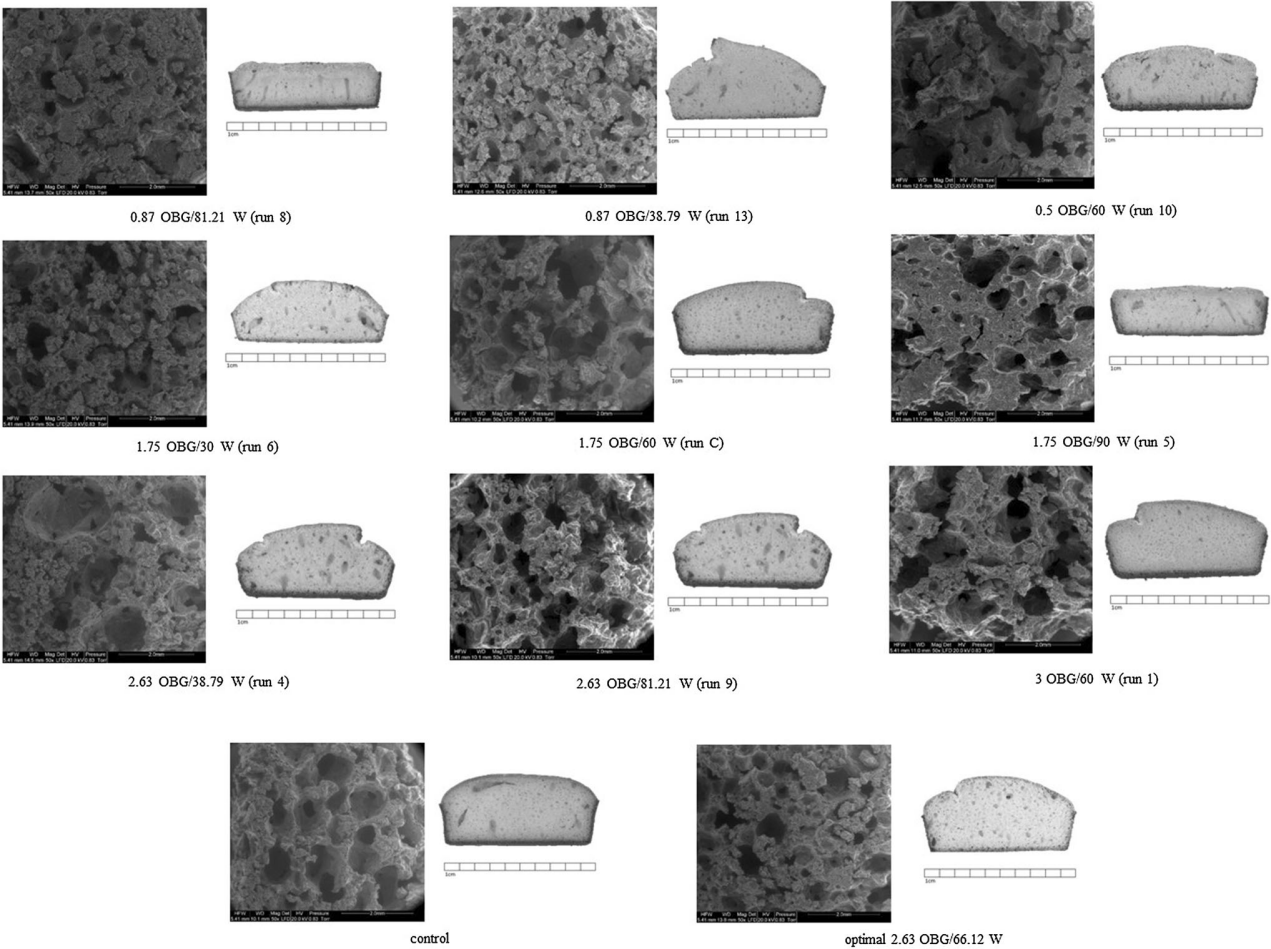
The microstructure of gluten-free yeast leavened cake under scanning electron microscopy and images of cake slices: *OBG* amount of oat β-glucan, *W* amount of water

The microstructure of the obtained GF cakes in each run of the experiment was analysed using scanning electron microscopy. In Fig. 2, the micrographs are presented at magnification 50. It can be seen that the control and optimised samples were comparable in terms of pore size and cake structure. These micrographs are the best confirmation of the structure-forming properties of oat β-glucan if the proportions of water and β-glucan are optimised. In general, the micrographs confirmed the rheological results. When the values of storage (G′) and loss (G″) modules were very low, the cake structure collapsed (0.87 OBG/81.21W, 1.75OBG/90W, 0.5OBG/60W). This means that the starch-protein network was too weak to maintain the structure, and the amount of β-glucan was too low to strengthen it. When the values of storage (G′) and loss (G″) were very high (higher than the control sample), the structure was too solid and pores could not be created because bubbles of CO_2_ and other volatile compounds did not manage to escape (0.87OBG/38.79, 1.78OBG/30W, 2.63OBG/38.79W). This means that the amount of water was not sufficient and the content of β-glucan was not enough to create structure. The results are comparable to the study of Turabi et al. (2010), where they compared different gums in gluten-free rice cakes. They also noticed that the addition of gum made the structure more porus, stable and that the volume of the cake had improved. Moreover, they also revealed that the higher apparent viscosity contributed to the better structure of the cake due to the improved formation of pores. The collapse of the structure was now less possible.

To complete microsturcture characterisation, the images of GF cake slices were captured. They are shown also in Fig. 2. Now, it can be easily seen how different levels of BG and W influened the structure of GF cake. The apperiance of the structure of the whole slice precisely corespond with amount of structure making agent and water. For example, the image of sample 0.87OBG/81.21W presents the collapsion of cake structure, while the image of optimal sample shows perfectly developed structure with porous area and volume comparable to control sample.

## Conclusion

The results showed that the optimisation process was crucial in order to develop a high-quality, gluten-free yeast leavened cake. The RSM was the proper tool to carry out and design the experiment. Thanks to RSM the evaluation of changes in results and the description of mechanism that occurred in GF batter and cake were possible. The study revealed that both oat β-glucan and water had the most significant impact on the gluten-free yeast leavened cake. Oat β-glucan affected not only specific volume and lightness which increased, but also difference in colour (ΔE) and firmness which decreased. Water had a significant impact on cooking yield, specific volume and lightness, which decreased. The quadratic term of this variable, however, showed that lightness and ΔE increased. The optimised amounts of water and oat β-glucan were 66.12% and 2.63% respectively. The final product showed their positive influence mostly on texture and sensory overall quality. The comparison of predicted and experimental values of chosen responses proved that the optimisation process and the model were properly fitted. Overall, the addition of oat β-glucan to gluten-free bakery products is possible when water content is well optimised. That is why, from a technological point of view, the application of oat β-glucan as a structure-making agent could be used in the food industry.
